# Identification of Key Genes and Pathways Associated with Oxidative Stress in Periodontitis

**DOI:** 10.1155/2022/9728172

**Published:** 2022-09-13

**Authors:** Zheng Zhang, Youli Zheng, Xiaowei Bian, Minghui Wang, Jiashu Chou, Haifeng Liu, Zuomin Wang

**Affiliations:** ^1^Tianjin Stomatological Hospital, School of Medicine, Nankai University, Tianjin 300000, China; ^2^State Key Laboratory of Natural and Biomimetic Drugs, Peking University, Beijing 100191, China; ^3^Tianjin Key Laboratory of Oral and Maxillofacial Function Reconstruction, Tianjin 300041, China; ^4^The School and Hospital of Stomatology, Tianjin Medical University, Tianjin 300070, China; ^5^Department of Stomatology, Beijing Chao-Yang Hospital, Capital Medical University, Beijing 100020, China

## Abstract

**Methods:**

The differentially expressed genes (DEGs) were identified using periodontitis-related microarray from the GEO database, and OS-genes were extracted from GeneCards database. The intersection of the OS-genes and the DEGs was considered as oxidative stress-related DEGs (OS-DEGs) in periodontitis. The Pearson correlation and protein-protein interaction analyses were used to screen key OS-genes. Gene set enrichment, functional enrichment, and pathway enrichment analyses were performed in OS-genes. Based on key OS-genes, a risk score model was constructed through logistic regression, receiver operating characteristic curve, and stratified analyses.

**Results:**

In total, 74 OS-DEGs were found in periodontitis, including 65 upregulated genes and 9 downregulated genes. Six of them were identified as key OS-genes (CXCR4, SELL, FCGR3B, FCGR2B, PECAM1, and ITGAL) in periodontitis. All the key OS-genes were significantly upregulated and associated with the increased risk of periodontitis. Functional enrichment analysis showed that these genes were mainly associated with leukocyte cell-cell adhesion, phagocytosis, and cellular extravasation. Pathway analysis revealed that these genes were involved in several signaling pathways, such as leukocyte transendothelial migration and osteoclast differentiation.

**Conclusion:**

In this study, we screened six key OS-genes that were screened as risk factors of periodontitis. We also identified multiple signaling pathways that might play crucial roles in regulating oxidative stress damage in periodontitis. In the future, more experiments need to be carried out to validate our current findings.

## 1. Introduction

Periodontitis is one of the most prevalent inflammatory conditions, characterized by bone and attachment destruction. This condition affects around 50% of the adult population worldwide and has now become the main cause of tooth loss in adults [1]. Periodontitis is a multifactorial disease, initiated by plaque bacteria that lead to excessive inflammation, breakdown of periodontal soft tissues, destruction of alveolar bone, and eventually tooth loss [2]. The development, progression, and aggressiveness of periodontal destruction depend on numerous environmental and host-related factors, both modifiable (for example, smoking) and nonmodifiable (for example, genetic susceptibility) [3]. Currently, the unequivocal mechanism that describes the development of periodontitis remains poorly understood, which makes it difficult for early prevention and control of the disease.

It is proposed that oxidative stress plays an important role in the pathogenesis of periodontitis [1]. Oxidative stress is a state of imbalance between oxidants and antioxidants production that results in the overproduction of reactive oxygen species and a comparative deficiency of antioxidants [4]. Reports from different studies have shown that patients with periodontitis have increased levels of oxidative stress markers in gingival crevicular fluid, saliva, and plasma [5]. Nonsurgical periodontal therapy has a beneficial influence on the levels of the antioxidant markers [6, 7]. Periodontitis-induced oxidative stress can trigger proinflammatory mechanisms and importantly osteoclastogenesis, which then leads to the bone loss that is observed in patients with periodontitis [8]. It has been reported that oxidative stress can activate NF-*κ*B signaling pathway to promote the expression of proinflammatory factors [9]. Antioxidant therapy can reduce oxidative stress damage and alleviate alveolar bone loss in periodontitis [5]. However, the exact pathophysiological mechanism involving oxidative stress is not yet fully explained in periodontitis.

Oxidative stress is generally regulated by differentially expressed oxidative stress-related genes (OS-genes) that are responsible, both directly and indirectly, for the pathogenesis of diseases [10, 11]. Up to now, only a small fraction of OS-genes has been studied intensively and is known to play an essential role in periodontitis progression [12]. Hence, identifying more key OS-genes may help validate the underlying mechanisms of periodontitis and offer therapeutic strategies for these patients. With the development of sequencing, bioinformatics analysis has been widely employed to identify the interaction between gene expression signatures and diseases. However, bioinformatics analysis of OS-genes has not been used to discover disease-specific biomarkers that correlate with periodontitis progression. Recently, large-scale genome profiles have provided gene expression data, which provides an excellent chance to identify potential OS-genes. Therefore, this study is aimed at finding out the key OS-genes in periodontitis from the point of view of bioinformatics analysis and providing a reference for further research of periodontitis.

## 2. Materials and Methods

### 2.1. Microarray Data

Two periodontitis-related gene expression profiles (GSE10334 and GSE16134) with a sample size greater than 10 were downloaded from the Gene Expression Omnibus (GEO) database (http://www.ncbi.nlm.nih.gov/geo/). Both GSE10334 and GSE16134 were based on the platform of GPL570 (Affymetrix Human Genome U133 Plus 2.0 Array). The two gene expression profiles included 424 periodontitis tissue samples and 133 normal tissue samples in total.

### 2.2. Differential Expression Analysis

The microarray data were normalized by the normalize quantiles function of the preprocess Core package in R software (version 3.4.1). Differential expression analysis was performed on the normalized datasets using the limma package (version 3.40.2). We set |log2 fold change (FC)| > log2 1.5 and *P* value < 0.05 as the thresholds for identifying differentially expressed genes (DEGs) in periodontitis.

### 2.3. Identification of OS-Genes

A total of 1119 OS-genes were extracted from GeneCards (https://www.genecards.org) with a relevance score ≥ 7. The intersection of the OS-genes selected from the GeneCards database and the DEGs from the periodontitis-related gene expression profiles was considered as oxidative stress-related differentially expressed genes (OS-DEGs) in periodontitis.

### 2.4. Gene Set Enrichment Analysis (GSEA) of OS-Genes

The WebGestalt online platform (http://www.webgestalt.org) was used for GSEA. The expression information of 1119 OS-genes in periodontitis was extracted from the gene expression profiles and imported into WebGestalt online platform. Normalized enrichment score was used to indicate the strength of the enrichment. The level of significance was defined at FDR ≤ 0.05.

### 2.5. Correlation Analysis of OS-DEGs

The correlation between every two OS-DEGs was analyzed via Pearson's correlation coefficient in GraphPad Prism 8.0.2. The web-based tools (http://www.bioinformatics.com.cn) were used for data visualization. As |*r*| values above 0.7 are statistically seen as showing a high level of correlation, we set *P* values < 0.05 and |*r*| > 0.7 as the thresholds for identifying paired genes in periodontitis.

### 2.6. Protein-Protein Interaction (PPI) Network Building and Hub OS-Gene Analysis

The upregulated and downregulated OS-DEGs were imported into the STRING database (http://www.string-db.org/) to obtain the PPI network. The PPI network was next imported into the Cytoscape software for visualization and analysis. The TOP 20 hub genes of the PPI network were identified as hub OS-genes using the cytoHubba tool.

### 2.7. Identification of Key OS-Genes

Top 20 OS-DEGs with the largest number of paired genes were then intersected with top 20 hub OS-genes, and the intersected genes were defined as key OS-genes. The relationship among key OS-genes was analyzed by Pearson's correlation coefficient and PPI network building.

### 2.8. Functional and Pathway Enrichment Analysis

To determine the biological processes and pathways of OS-genes in periodontitis, ClusterProfiler R package was used for Gene Ontology (GO) biological processes and Kyoto Encyclopedia of Genes and Genomes (KEGG) pathway enrichment analysis. The GO analysis included three categories: biological process, molecular function, and cellular component. *P* value < 0.05 was considered as statistically significant. KEGG database was used to view pathways class.

### 2.9. Relevance Analysis of Key OS-Genes and Oxidative Stress Biomarkers

The relationships between key OS-genes and oxidative stress biomarkers, including eight oxidative biomarkers and five antioxidant biomarkers, were expressed by relevance scores. These relevance scores were obtained from GeneCards and imported into GraphPad Prism 8.0.2 for visualization. The biomarkers with a relevance score ≥ 7 were selected to construct the network of key OS-genes and oxidative stress biomarkers using Cytoscape software.

### 2.10. Risk Evaluation of Key OS-Genes

Univariate logistic regression analysis was used to calculate the odds ratio (OR) and 95% confidence interval (CI) for the association between key OS-genes expression and the risk of periodontitis. Multivariate logistic regression analysis was used to screen independent variables among key OS-genes, and receiver operating characteristic (ROC) curve was performed to study their essential effect on the disease. The risk score formula for each patient was constructed based on the estimated regression coefficient value of key OS-genes in multivariate logistic regression.

## 3. Results

### 3.1. Recognition of DEGs of Periodontitis

**Figure 1:**
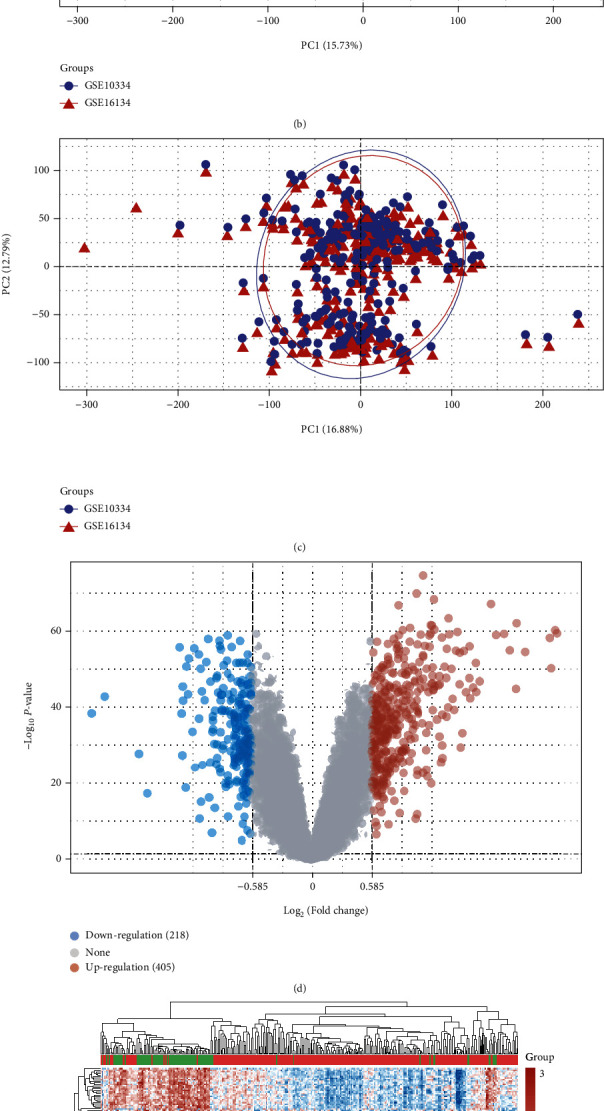
Identification of differentially expressed genes (DEGs) in periodontitis. (a) The boxplot of the normalized data. (b) PCA results before batch removal for multiple datasets. (c) PCA results after batch removal. (d) The volcano plots of the DEGs. (e) The heatmap of the DEGs.

The RNA expression profile datasets (GSE16134 and GSE10334) were normalized as shown in Figure 1(a). The differences between samples were significantly reduced after batch correction (Figures 1(b) and 1(c)). A total of 623 DEGs were identified, including 405 upregulated and 218 downregulated genes (Figure 1(d)). Additionally, a heatmap of the DEGs is shown Figure 1(e).

### 3.2. GSEA of OS-Genes

**Figure 2:**
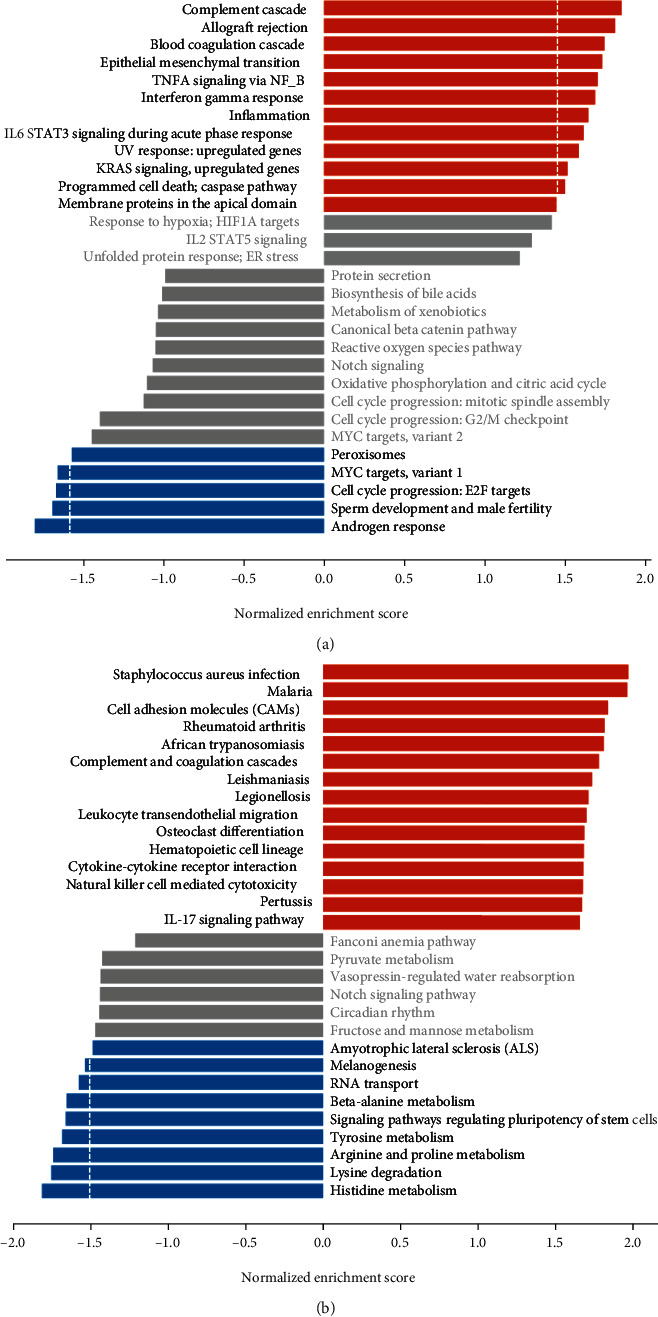
Gene set enrichment analysis (GSEA) of 1119 oxidative stress-related genes (OS-genes). (a) Hallmark gene set database. (b) KEGG gene set database.

The expression information of 1119 OS-genes was used to perform GSEA. The Hallmark gene set database showed that most of the upregulated genes were involved in epithelial mesenchymal transition, TNFA signaling via NF-*κ*B, inflammation, interferon gamma response, and IL6 STAT3 signaling during acute phase response. The downregulated genes were involved in androgen response, MYC targets, variant 1, and peroxisomes (Figure 2(a)). The KEGG gene set database demonstrated that the upregulated genes were involved in osteoclast differentiation, IL-17 signaling pathway, cytokine-cytokine receptor interaction, leukocyte transendothelial migration, and cell adhesion molecules. The downregulated genes were involved in histidine metabolism, lysine degradation, arginine and proline metabolism, tyrosine metabolism, and signaling pathways regulating pluripotency of stem cells (Figure 2(b)).

### 3.3. Identification and Enrichment Analysis of OS-DEGs

**Figure 3:**
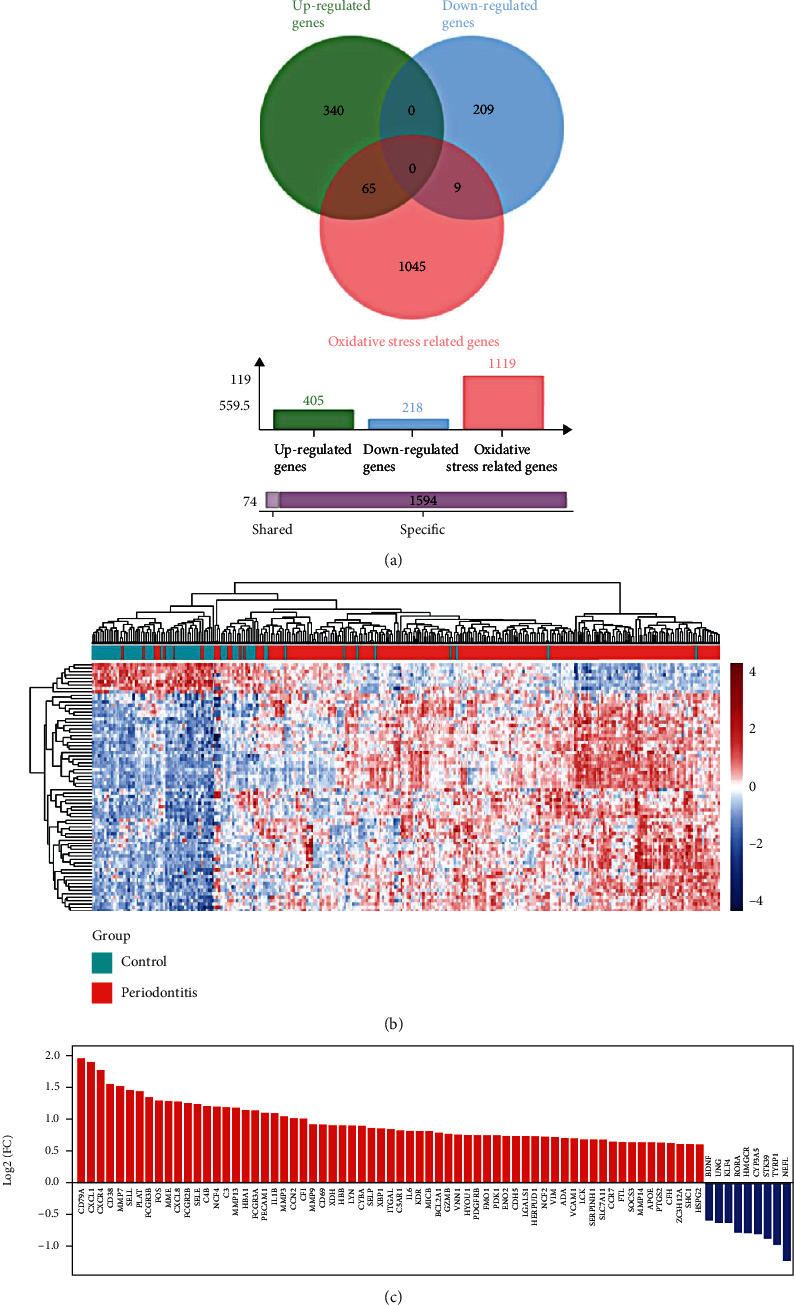
Identification of oxidative stress-related differentially expressed genes (OS-DEGs). (a) Venn diagram of the intersection of oxidative stress-related genes (OS-genes) and differentially expressed genes (DEGs) in periodontitis. (b) Heatmap of the OS-DEGs. (c) Histogram of the OS-DEGs.

After conducting a combined analysis of OS-genes and DEGs of periodontitis, 74 genes were screened out as OS-DEGs in periodontitis, including 65 upregulated genes and 9 downregulated genes (Figure 3(a)). The distribution of OS-DEGs is shown in Figures 3(b) and 3(c).

**Figure 4:**
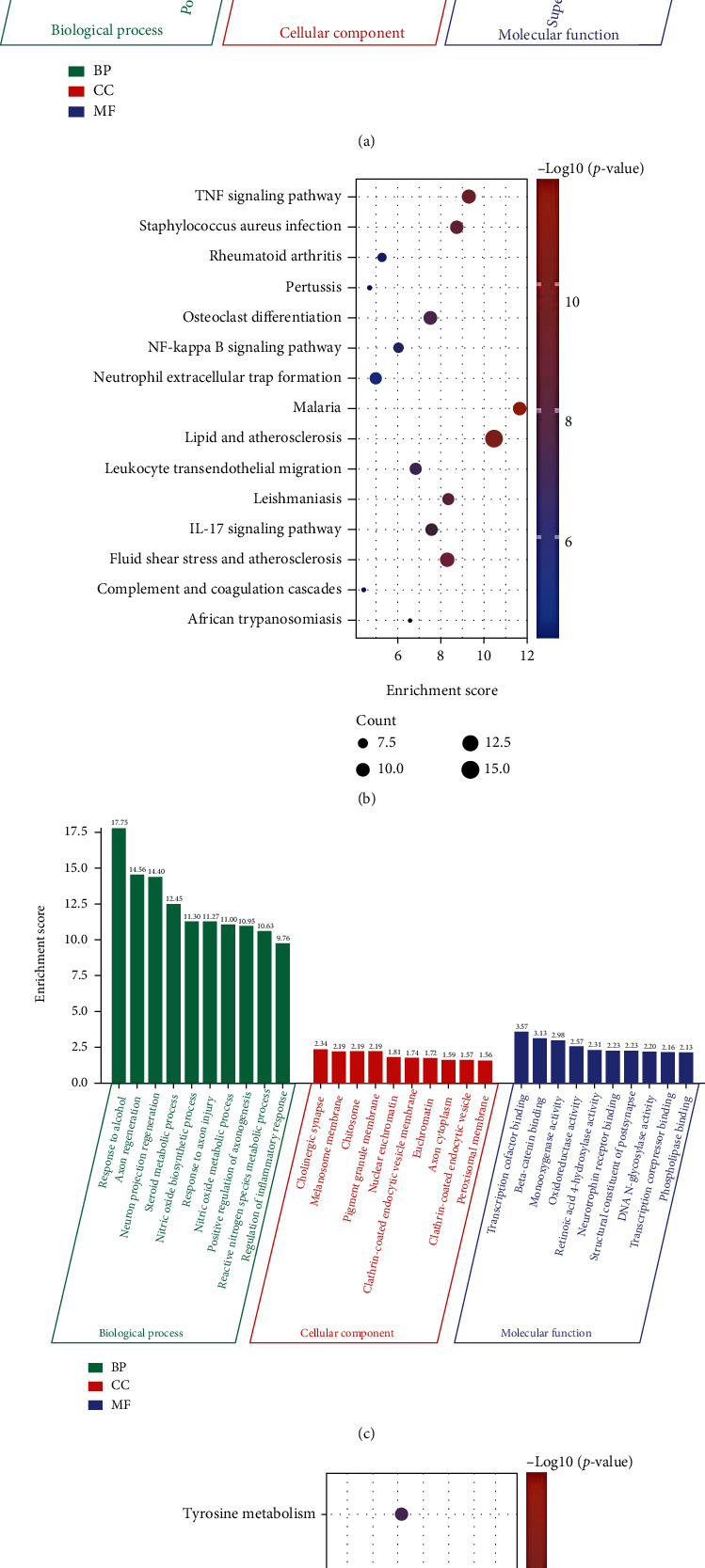
Enrichment analysis of oxidative stress-related differentially expressed genes (OS-DEGs). (a) The top 10 lists of GO enrichment analysis of upregulated OS-DEGs. (b) The top 15 lists of KEGG pathway enrichment analysis of upregulated OS-DEGs. (c) The top 10 lists of GO enrichment analysis of downregulated OS-DEGs. (d) The lists of KEGG pathway enrichment analysis of downregulated OS-DEGs.

GO enrichment analysis revealed that upregulated OS-DEGs were significantly enriched in response to oxidative stress, response to molecule of bacterial origin, leukocyte cell-cell adhesion, response to lipopolysaccharide, regulation of leukocyte cell-cell adhesion, positive regulation of cell activation, and regulation of inflammatory response (Figure 4(a) and Supplementary Figure S1-S3). KEGG pathway enrichment analysis showed that these genes were significantly enriched in TNF signaling pathway, IL-17 signaling pathway, osteoclast differentiation, and NF-kappa B signaling pathway (Figure 4(b) and Supplementary Figure S4).

GO enrichment analysis demonstrated that downregulated OS-DEGs were significantly enriched in nitric oxide biosynthetic process, nitric oxide metabolic process, reactive nitrogen species metabolic process, and regulation of inflammatory response (Figure 4(c)). The enriched pathways for these genes were base excision repair, tyrosine metabolism, and primary immunodeficiency (Figure 4(d)).

### 3.4. Correlation Analysis of OS-DEGs

**Figure 5:**
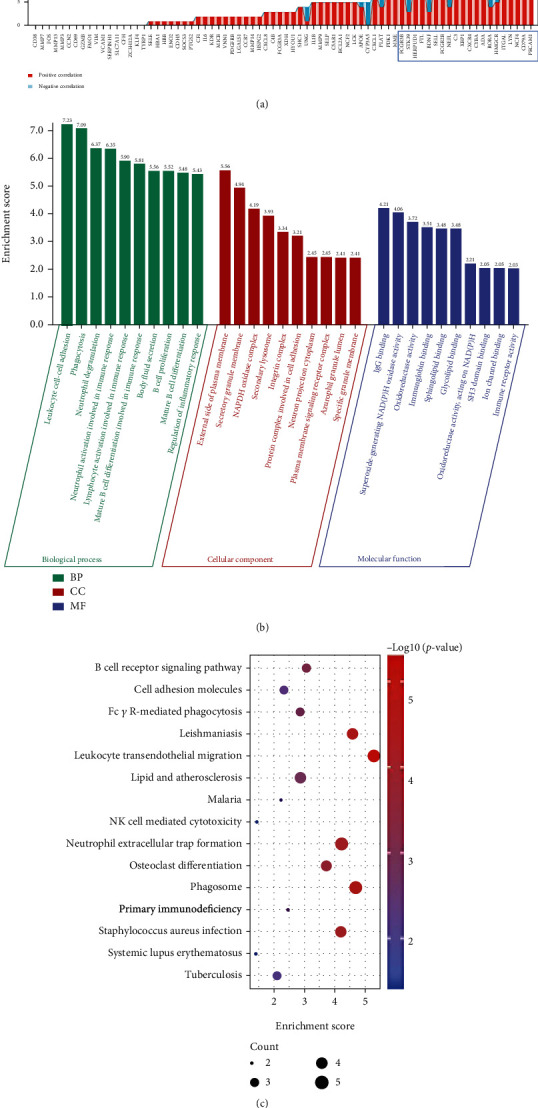
Identification and enrichment analysis of the paired genes. (a) Top 20 paired genes were screened from the oxidative stress-related differentially expressed genes (OS-DEGs). (b) The top 10 lists of GO enrichment analysis of the top 20 paired genes. (c) The top 15 lists of KEGG pathway enrichment analysis of the top 20 paired genes.

A total of 203 pairs of OS-DEGs were identified in periodontitis (*P* values < 0.05, |*r*| > 0.7) (Supplementary Figure S5). As shown in Figure 5(a), PECAM1, CD79A, and NCF4 possessed more than 20 paired genes. SELL, FCGR2B, NEFL, C3, XBP1, CXCR4, CYBA, ADA, RORA, HMGCR, ITGAL, and LYN possessed more than 10 paired genes.

GO enrichment results showed that top 20 OS-DEGs with the largest number of paired genes were involved in phagocytosis, regulation of inflammatory response, and immune response (Figure 5(b) and Supplementary Figure S6-S8). KEGG enrichment results demonstrated that these genes were associated with B cell receptor signaling pathway, cell adhesion molecules, leukocyte transendothelial migration, neutrophil extracellular trap formation, and osteoclast differentiation (Figure 5(c) and Supplementary Figure S9).

### 3.5. Identification and Enrichment Analysis of Hub OS-Genes

**Figure 6:**
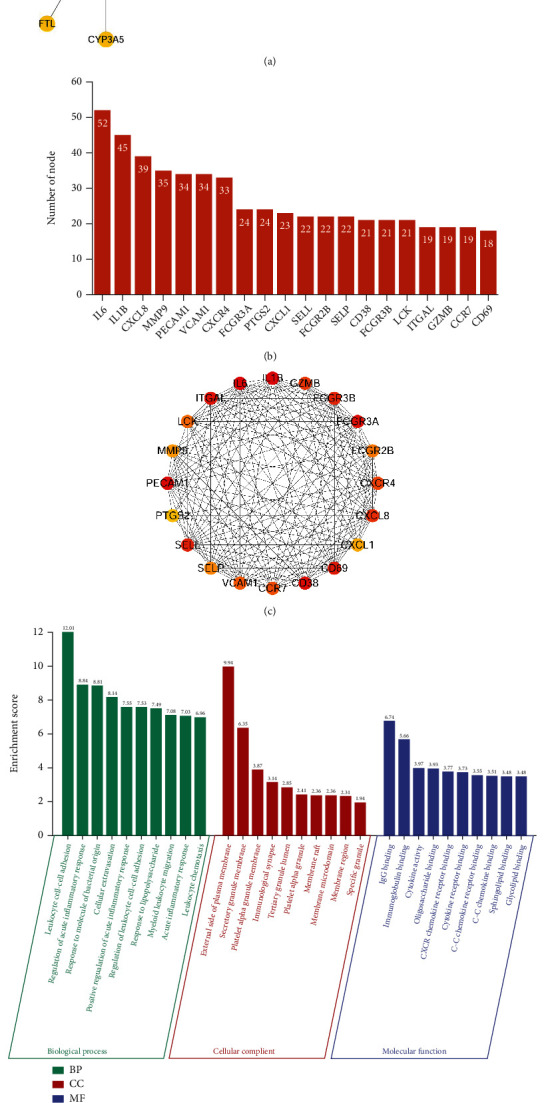
The protein-protein interaction (PPI) network and hub gene analyses. (a) The PPI network of the oxidative stress-related differentially expressed genes (OS-DEGs). (b) The connectivity rank of genes. (c) The relationship between hub OS-genes. (d) The top 10 lists of GO enrichment analysis of the hub OS-genes. (e) The top 15 lists of KEGG pathway enrichment analysis of the hub OS-genes.

The PPI network of the OS-DEGs was built according to the STRING database, including 74 nodes and 509 edges (Figure 6(a)). The targets were sorted by target connectivity from large to small in the PPI network; the top 20 are shown in Figure 6(b). Using cytoHubba, we obtained the top 20 hub OS-genes, including IL6, IL1B, PECAM1, CD38, FCGR3A, ITGAL, CD69, SELL, FCGR3B, CXCL8, GZMB, CXCR4, CCR7, VCAM1, LCK, FCGR2B, SELP, MMP9, CXCL1, and PTGS2 (Figure 6(c)).

Figure 6(d) and Supplementary Figure S10-S12 showed that top 20 hub OS-genes were mainly enriched in several biological processes, for example, leukocyte cell-cell adhesion, regulation of acute inflammatory response, response to molecule of bacterial origin, and response to lipopolysaccharide.  Figure 6(e) and Supplementary Figure S13 show that top 20 hub OS-genes were mainly enriched in several pathways, such as IL-17 signaling pathway, NF-*κ*B signaling pathway, TNF signaling pathway, and osteoclast differentiation.

### 3.6. Identification of Key OS-Genes

**Figure 7:**
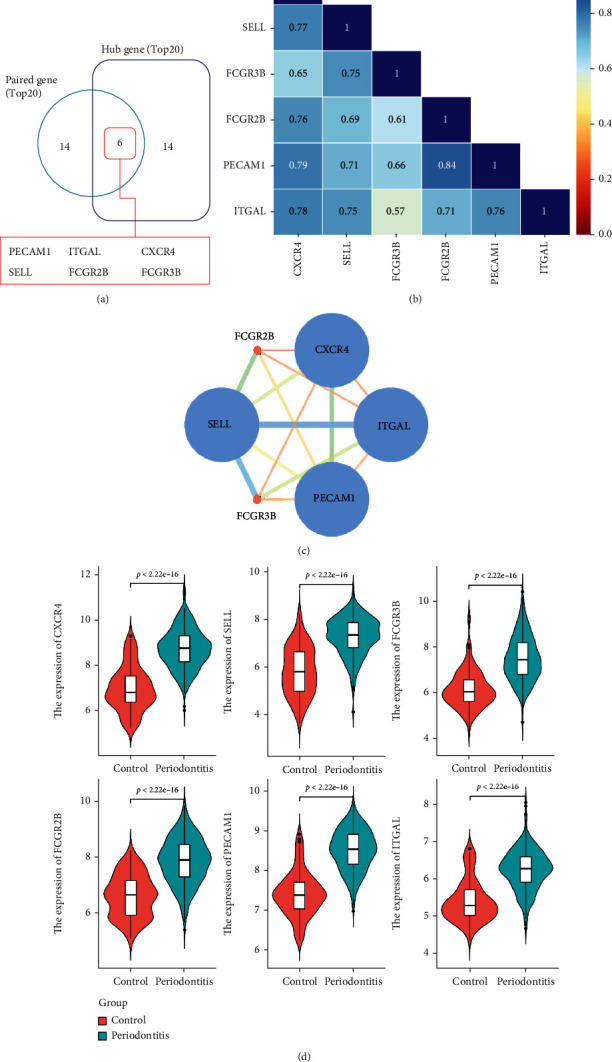
Identification of key oxidative stress-related genes (OS-genes). (a) Venn diagram of the intersection of OS-DEGs paired genes and hub OS-genes. (b) Heatmap of the correlation among key OS-genes. (c) The PPI network of the key OS-genes. (d) The expression levels of the key OS-genes.

The intersection of top 20 hub OS-genes and top 20 paired OS-genes revealed six key OS-genes, including CXCR4, SELL, FCGR3B, FCGR2B, PECAM1, and ITGAL (Figure 7(a)). There was a significantly positive correlation among the key OS-genes (Figure 7(b)). The key OS-genes in PPI network were closely linked and could act as a whole (Figure 7(c)). All of the six key OS-genes were upregulated in periodontitis (Figure 7(d)).

### 3.7. Functional Enrichment Analysis of Key OS-Genes

**Figure 8:**
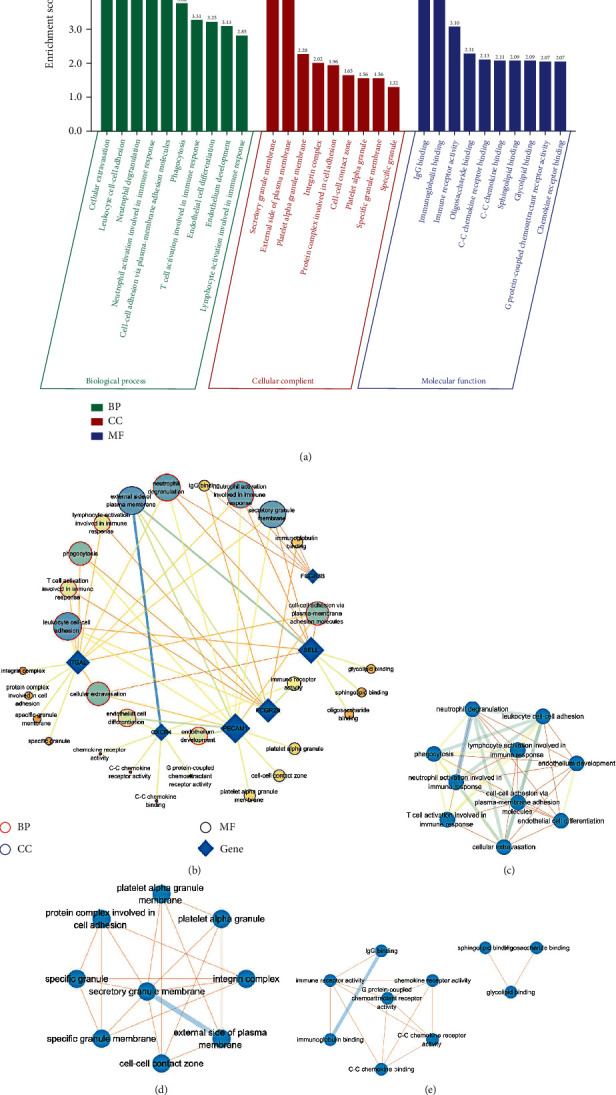
GO enrichment analysis of key oxidative stress-related genes (OS-genes). (a) The top 10 lists of GO enrichment analysis of the key OS-genes. (b) The networks of key OS-genes with GO terms. (c–e) The relation of biological processes (c), molecular function (d), and cellular component (e) involved in key OS-genes.

For GO enrichment analysis, key OS-genes were significantly enriched in leukocyte cell-cell adhesion, neutrophil degranulation, phagocytosis, and many immune responses (Figure 8(a)). The networks of key OS-genes with GO terms showed that PECAM1, SELL, and ITGAL commonly regulated cell-cell adhesion via plasma-membrane adhesion molecules, cellular extravasation, leukocyte cell-cell adhesion, neutrophil activation involved in immune response, and neutrophil degranulation (Figure 8(b)).

GO networks analyses revealed that leukocyte cell-cell adhesion, neutrophil degranulation, and neutrophil activation involved in immune response were the main biological processes involved in key OS-genes (Figure 8(c)). Secretory granule membrane was the most significantly enriched cellular component (Figure 8(d)), and immune receptor activity was the main molecular function associated with key OS-genes (Figure 8(e)).

### 3.8. Pathway Enrichment Analysis of Key OS-Genes

**Figure 9:**
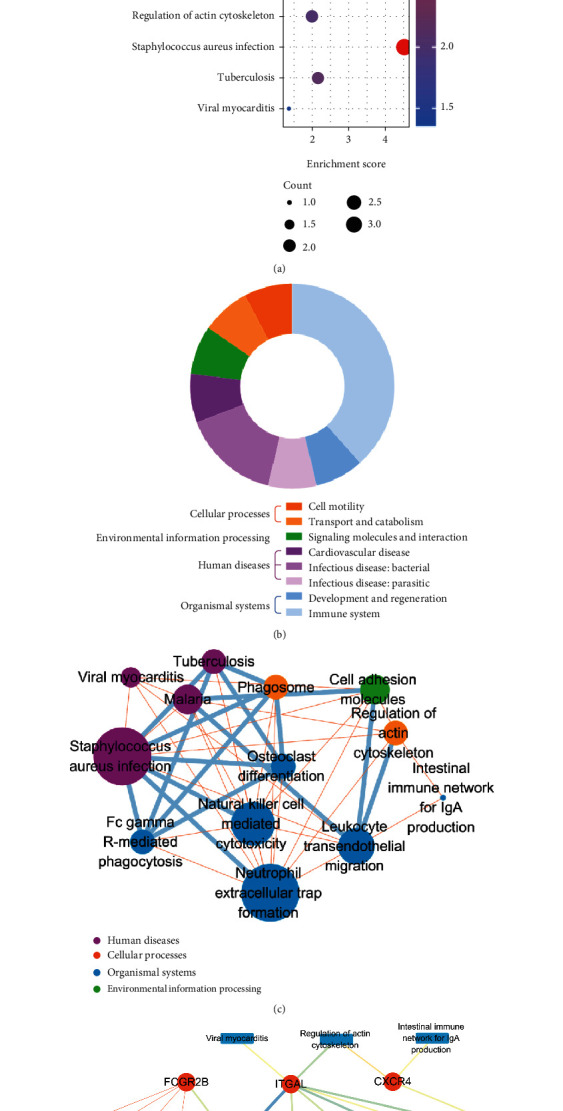
KEGG pathway enrichment analysis of key oxidative stress-related genes (OS-genes). (a) The lists of KEGG pathway enrichment analysis of the key OS-genes. (b) The pathways' class counts. (c) The relation of KEGG pathways. (d) The networks of key OS-genes with KEGG pathways.

KEGG pathway enrichment analysis for key OS-genes revealed that they were significantly enriched in leukocyte transendothelial migration, cell adhesion molecules, Fc gamma R-mediated phagocytosis, osteoclast differentiation, and neutrophil extracellular trap formation (Figure 9(a)).  Figure 9(b) revealed that these pathways were primarily involved in “infectious diseases” and “immune system.”

KEGG networks analyses showed that neutrophil extracellular trap formation, Staphylococcus aureus infection, natural killer cell mediated cytotoxicity, leukocyte transendothelial migration, and osteoclast differentiation were the main pathways involved in key OS-genes (Figure 9(c)). The networks of key OS-genes with KEGG pathways demonstrated that PECAM1, ITGAL, and CXCR4 commonly affected leukocyte transendothelial migration. PECAM1 together with ITGAL and SELL participated in cell adhesion molecules (Figure 9(d)).

### 3.9. The Relevance Analysis of Key OS-Genes and Oxidative Stress Biomarkers

**Figure 10:**
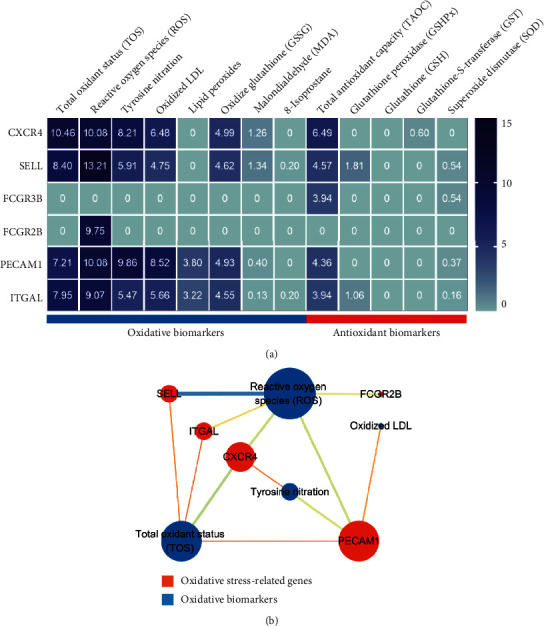
The relevance analysis of key OS-genes and oxidative stress biomarkers. (a) Heatmap of the relevance score of each key OS-genes. (b) The network of key OS-genes with oxidative stress biomarkers.

GeneCards database demonstrated that six key OS-genes were mainly related to oxidative biomarkers, but not to antioxidant biomarkers, the relevance scores are shown in Figure 10(a). The networks of key OS-genes with oxidative stress biomarkers revealed that five out of six key OS-genes, such as CXCR4, SELL, FCGR2B, PECAM1, and ITGAL, were associated with reactive oxygen species (ROS). Four key OS-genes (CXCR4, SELL, PECAM1, and ITGAL) were involved in total oxidant status (TOS) (Figure 10(b)).

### 3.10. Risk Evaluation of Key OS-Genes

**Figure 11:**
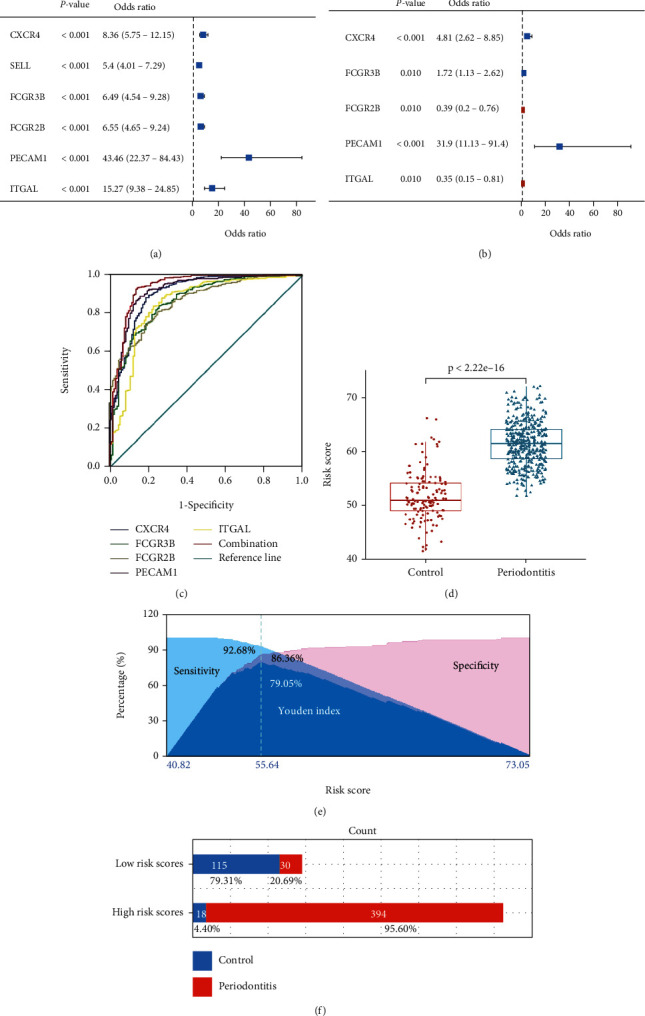
Risk evaluation of key OS-genes. (a) Univariate logistic regression analysis of key OS-genes in periodontitis. (b) Screening of independent variables among key OS-genes using multivariate logistic regression analysis (backward). (c) The ROC curve of key OS-genes in periodontitis. (d) The comparison of risk score between periodontitis and control. (e) The Youden index and corresponding optimal cut-point for risk score. (f) The percent of periodontitis patients in the high-risk and the low-risk groups.

To determinate the association between key OS-genes expression and the risk of periodontitis, we conducted univariate logistic regression analysis. As shown in Figure 11(a), all six key OS-genes were associated with increased risk of periodontitis. Multivariate logistic regression analysis was used to screen independent variables among key OS-genes, and CXCR4, FCGR3B, FCGR2B, PECAM1, and ITGAL were finally screened (Figure 11(b)). Then ROC curves were next established for these genes. The area under the curve (AUC) for CXCR4, FCGR3B, FCGR2B, PECAM1, and ITGAL was 0.91, 0.87, 0.87, 0.92, and 0.85, respectively. When these genes were combined, the AUCs increased to 0.94 (Figure 11(c)).

The risk score (Risk Score = CXCR4 × 2.91 + FCGR2B × (−1.75) + PECAM1 × 6.41 + ITGAL × (−1.93) + FCGR3B × 1) was obtained by logistic regression analysis. As shown in Figure 11(d), the risk score was significantly higher in periodontitis patients compared with the controls. Based on the Youden index, patients were allocated into the high-risk and low-risk groups (Figure 11(e)). The percent of periodontitis patients in the high-risk group (95.60%) was significantly higher than that (20.69%) in the low-risk group (Figure 11(f)).

## 4. Discussion

In recent years, increasing evidence has shown that oxidative stress plays an important role in the pathogenesis of various types of chronic inflammation, including periodontitis [13]. It has been demonstrated previously that protecting periodontal tissues or cells from oxidative stress by blocking OS-gene activation in inflammation can reduce periodontal tissue loss [12]. Although many publications have reported on oxidative stress biomarker levels in patients with periodontitis, very few studies evaluate the OS-genes in the pathogenesis of periodontitis [8]. In the present study, by performing multiple bioinformatics analysis methods, we firstly identified six key OS-genes (CXCR4, SELL, FCGR3B, FCGR2B, PECAM1, and ITGAL) in periodontitis, whose expression levels were significantly upregulated. Moreover, several pathways such as osteoclast differentiation and leukocyte transendothelial migration may be the potential mechanisms of key OS-genes in the pathogenesis of periodontitis.

Periodontitis is a chronic infectious disease in which the periodontal bacteria initiate the host immune response leading to the destruction of the periodontal tissue [14]. In other words, infection of periodontal bacteria and host immunity jointly contribute to the pathological processes of the periodontal destruction. In this study, the results of GSEA showed that these OS-genes were mainly involved in inflammation, interferon gamma response. GO enrichment analysis showed that the OS-DEGs were mainly enriched in terms that were related to infection and immune response, such as response to molecule of bacterial origin, response to lipopolysaccharide, leukocyte cell-cell adhesion, and regulation of leukocyte cell-cell adhesion. Interestingly, the hub OS-genes have been also found to be associated with the above biological processes. These results indicate that OS-genes play a critical role in various stages of periodontitis progression.

Up to date, numerous studies have revealed several signaling pathways involved in the development of periodontitis, such as NF-*κ*B signaling pathway [15], IL-17 signaling pathway [16], and Wnt/*β*-catenin signaling pathway [17]. Our previous study has shown that NF-*κ*B signaling is involved in periodontal ligament stem cells osteogenesis following inflammatory stimulation [18]. IL-17 signaling pathway is related to the metabolism of the alveolar bone [16]. In our study, GSEA results showed that the OS-genes in periodontitis mainly participated in leukocyte transendothelial migration, osteoclast differentiation, and IL-17 signaling pathway. This finding is according with our results from KEGG pathways enriched analysis of OS-DEGs and hub OS-genes. Besides, NF-*κ*B signaling pathway is another mechanism associated with these genes. In addition, the top 20 paired OS-genes and key OS-genes have been also found to be associated with leukocyte transendothelial migration and osteoclast differentiation. These findings suggest that such signaling pathways may be an important mechanism of OS-genes in the pathogenesis of periodontitis.

Platelet endothelial cell adhesion molecule-1 (PECAM1), also termed CD31, is a member of the immunoglobulin gene superfamily of cell adhesion molecules [19]. PECAM-1 is vital to the regulation of inflammatory responses, and inhibition of PECAM1 has been documented to alleviate symptoms of several inflammatory diseases such as arthritis, atherosclerosis, and pulpitis [20]. Furthermore, PECAM1 has been identified a potential biomarker for periodontitis diagnosis and prognosis [21]. The current study found that PECAM-1 was significantly upregulated in periodontitis tissues compared to normal tissues, and possessed up to 25 paired genes in 74 OS-DEGs. One of PECAM-1's most prominent functions is its role in mediating the final steps of transendothelial migration of leukocytes across endothelial cells [22]. In addition, we found that PECAM-1 was significantly associated with endothelial cell differentiation, endothelium development, and leukocyte cell-cell adhesion. The present data indicate that PECAM1 may play a central role in the pathogenesis of periodontitis partially through exerting effects on both leukocytes and endothelial cells.

Our other key OS-genes, C-X-C chemokine receptor type 4 (CXCR4) is a 352 amino acid rhodopsin-like *G* protein-coupled receptors [23]. Recently, it was found that CXCR4 plays a key role in mediating oxidative stress-induced podocyte damage, proteinuria, and glomerulosclerotic lesions [24]. Interestingly, CXCR4 neutralization in periodontal inflammation has been shown to significantly suppress alveolar bone resorption [25]. Moreover, previous study also suggested that CXCR4 can inhibit nitric oxide release from infiltrating macrophages and is involved in modulation of the mechanical sensitivity in the periodontal tissue in periodontitis [26]. In this study, CXCR4 was found to be one of the most highly overexpressed key OS-genes in periodontitis tissues. It could regulate chemokine receptor activity, response to chemokine, C-C chemokine receptor activity, and C-C chemokine binding. Therefore, we can reasonably speculate that the chemotactic activity may account for the effects of CXCR4 in the pathogenesis of periodontitis.

Importantly, the six key OS-genes not only work alone but also have connections with each other. For example, PECAM1 can combine with CXCR4 to trigger inflammatory cell infiltration and inflammation progression [20]. Both PECAM1and SELL are pro angiogenic genes, their physiological interactions account for the pathogenesis of chronic rhinosinusitis [27]. Moreover, SELL together with ITGAL is related to cell adhesion and migration [28]. Our PPI network and enrichment analysis show that PECAM1 and CXCR4 may interact with each other, and are commonly enriched in endothelial cell differentiation and endothelium development. CXCR4 may interact with PECAM1, and both of them participate in leukocyte transendothelial migration. Our analysis indicates that the combined effects of the six key OS-genes on periodontitis are more significant than a single gene.

## 5. Conclusions

In conclusion, through a series of bioinformatics analysis, we finally screened six key OS-genes that are significantly associated with the increased risk of periodontitis. We also identified multiple signaling pathways that might play crucial roles in regulating oxidative stress in periodontitis. This study provides novel research targets for studying the pathogenesis and progression of patients with periodontitis.

## Data Availability

The data of the findings in this study are available from the corresponding author upon reasonable request.

## Supplementary Materials

Supplementary Materials The supplementary material containing Supplementary Figure S1-S13 and figure legends are available in supplementary files.
