# Supplementary material for: Identification of Key Genes and Pathways Associated with Oxidative Stress in Periodontitis
Source: Oxid Med Cell Longev. 2022 Sep 13;2022:9728172. doi: 10.1155/2022/9728172 (PMC9489423; doi:10.1155/2022/9728172)
Supplement: Supplementary Materials — The supplementary material containing Supplementary Figure S1-S13 and figure legends are available in supplementary files. [file 9728172.f1.pdf]

Identification of Key Genes and Pathways Associated with Oxidative Stress in Periodontitis

Zheng Zhang [1, 2, 3], Youli Zheng [4], Xiaowei Bian [4], Minghui Wang [4], Jiashu Chou [1], Haifeng Liu [1,3], and Zuomin Wang [5]

[1] *Tianjin Stomatological Hospital, School of Medicine, Nankai University, Tianjin, 300000, China*

[2] *State Key Laboratory of Natural and Biomimetic Drugs, Peking University, Beijing 100191, China*

[3] *Tianjin Key Laboratory of Oral and Maxillofacial Function Reconstruction, Tianjin 300041, China*

[4] *The School and Hospital of Stomatology, Tianjin Medical University, Tianjin 300070, China*

[5] *Department of Stomatology, Beijing Chao-Yang Hospital, Capital Medical University, Beijing 100020, China*

Zheng Zhang, Youli Zheng and Xiaowei Bian contributed equally in this study.

Correspondence should be addressed to **Zuomin Wang**; wzuomin@sina.cn and **Haifeng Liu**; lhaifeng2006@sina.com

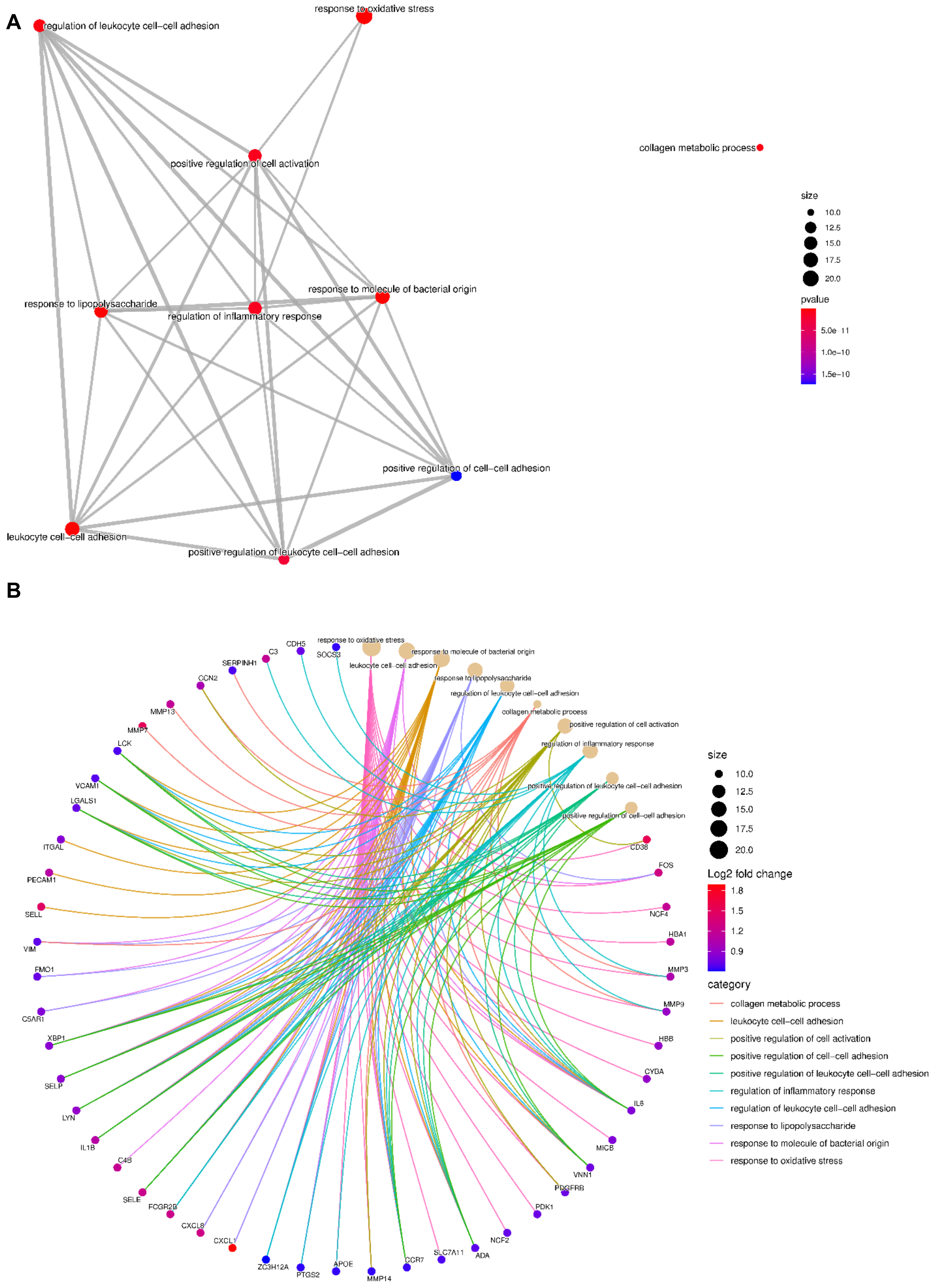

Supplementary Figure S1 (A) The relation of biological processes involved in up-regulated OS-DEGs; (B) The networks of up-regulated OS-DEGs with biological processes.

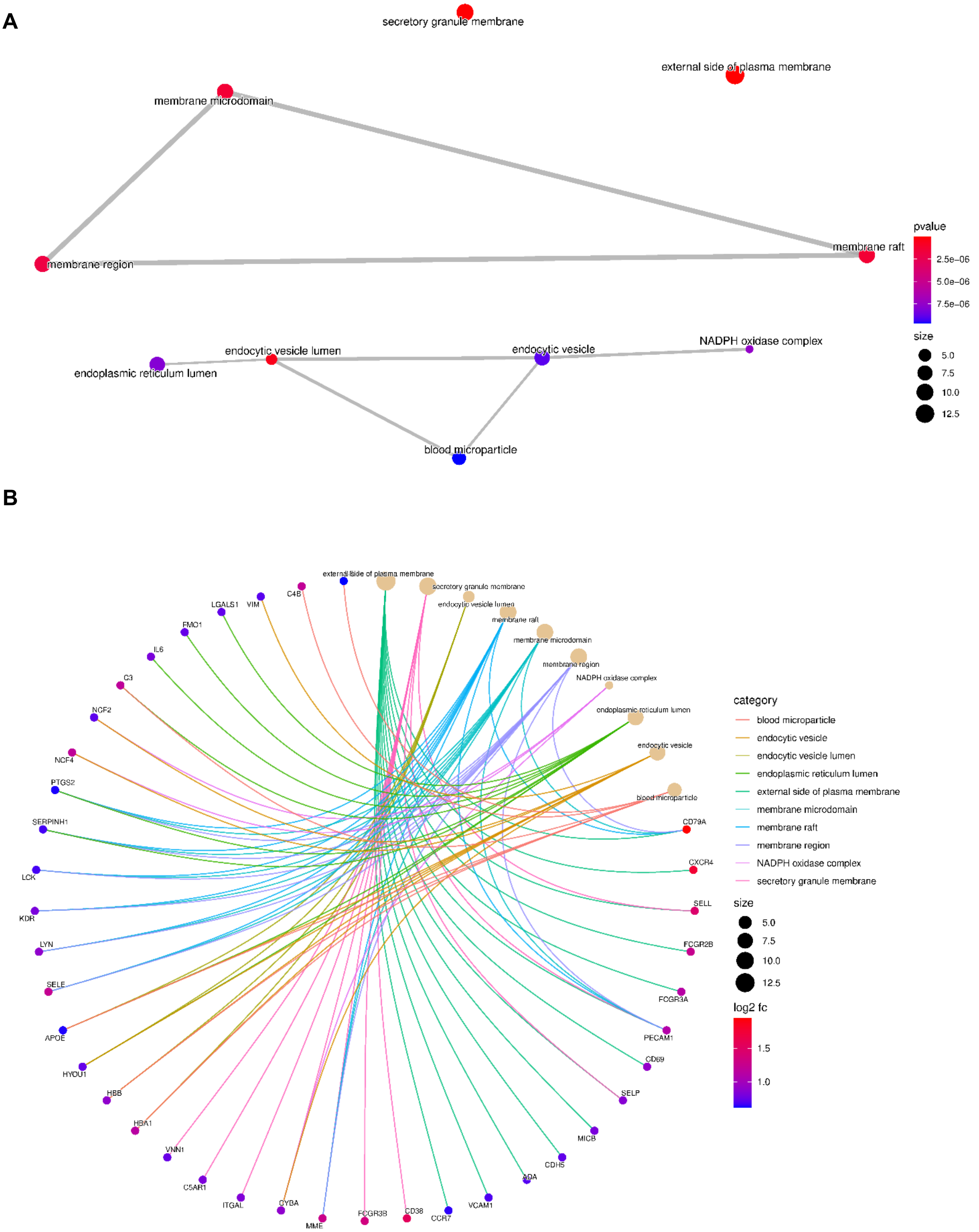

Supplementary Figure S2 (A) The relation of cellular components involved in up-regulated OS-DEGs; (B) The networks of up-regulated OS-DEGs with cellular components.

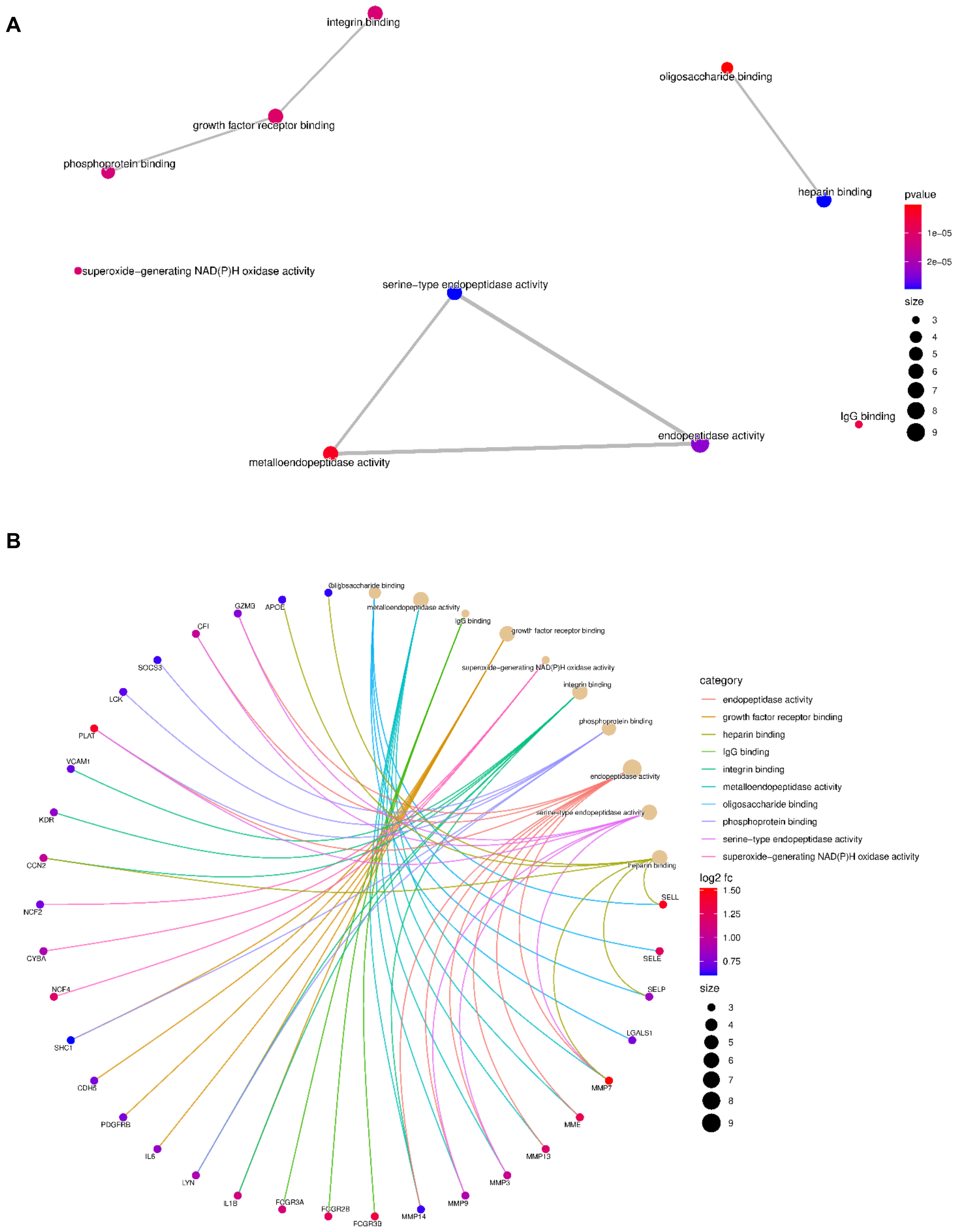

Supplementary Figure S3 (A) The relation of molecular functions involved in up-regulated OS-DEGs; (B) The networks of up-regulated OS-DEGs with molecular functions.

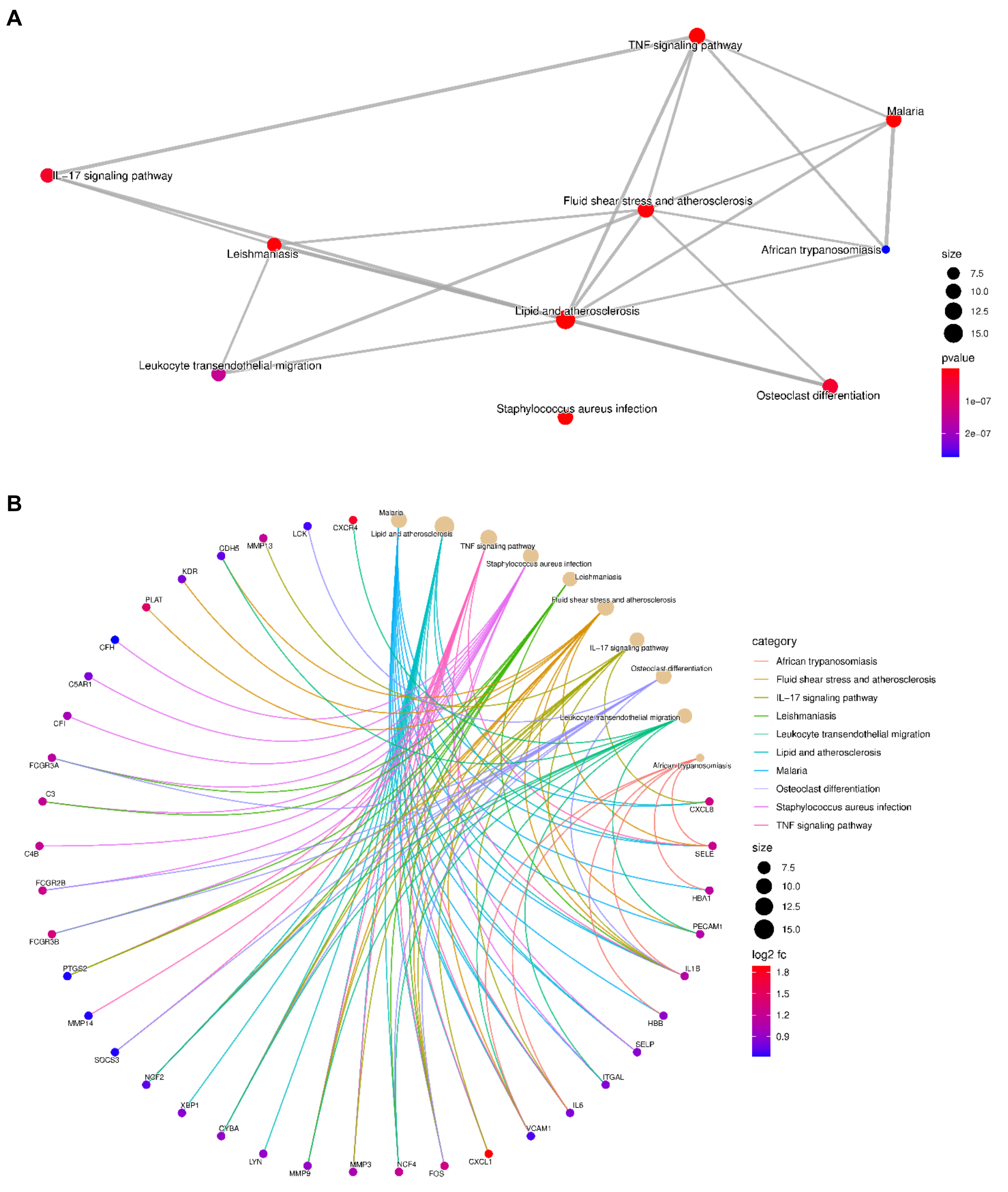

Supplementary Figure S4 (A) The relation of KEGG pathways involved in up-regulated OS-DEGs; (B) The networks of up-regulated OS-DEGs with KEGG pathways.

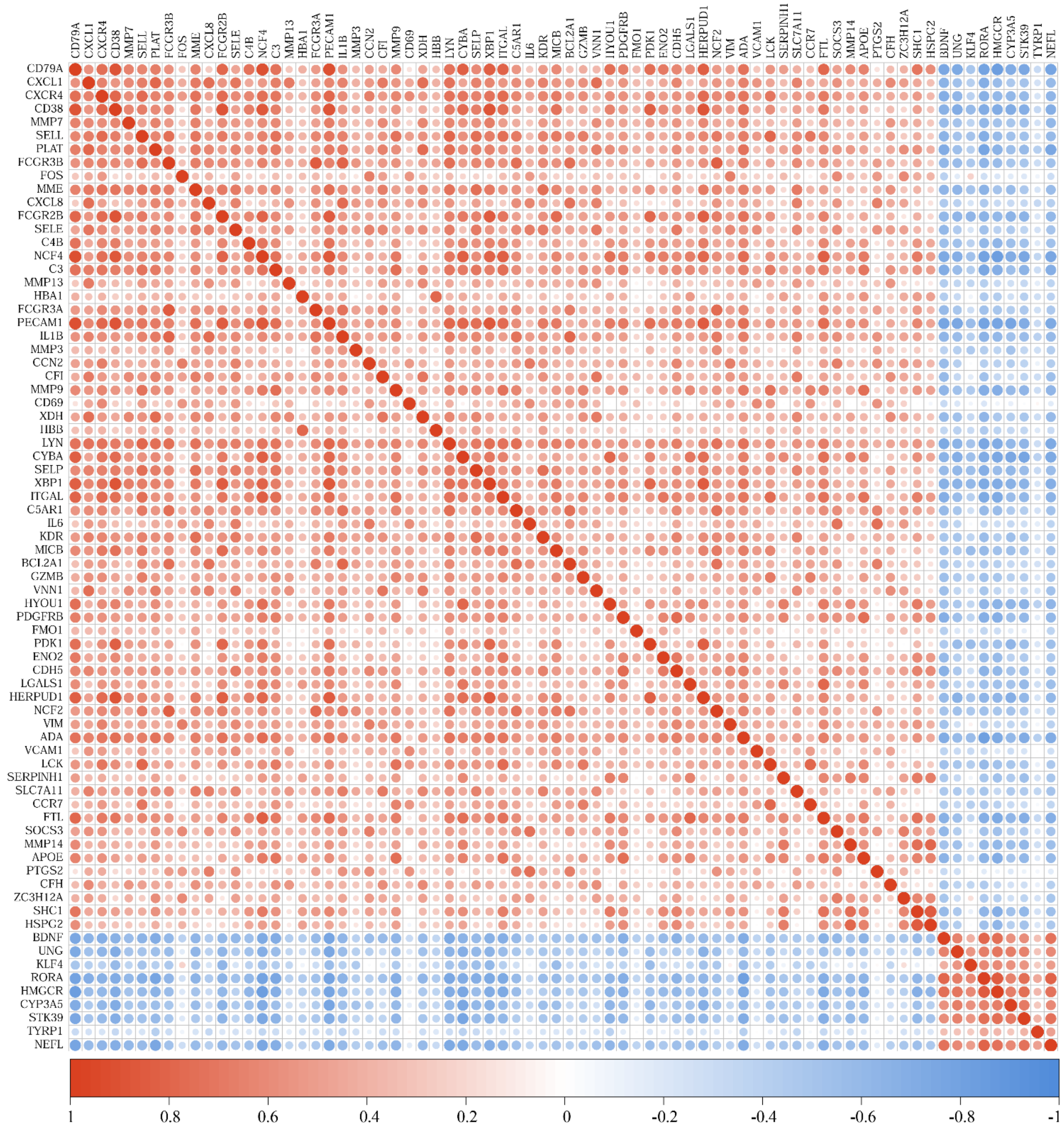

Supplementary Figure S5 Heatmap of the correlation among oxidative stress-related differential expressed genes

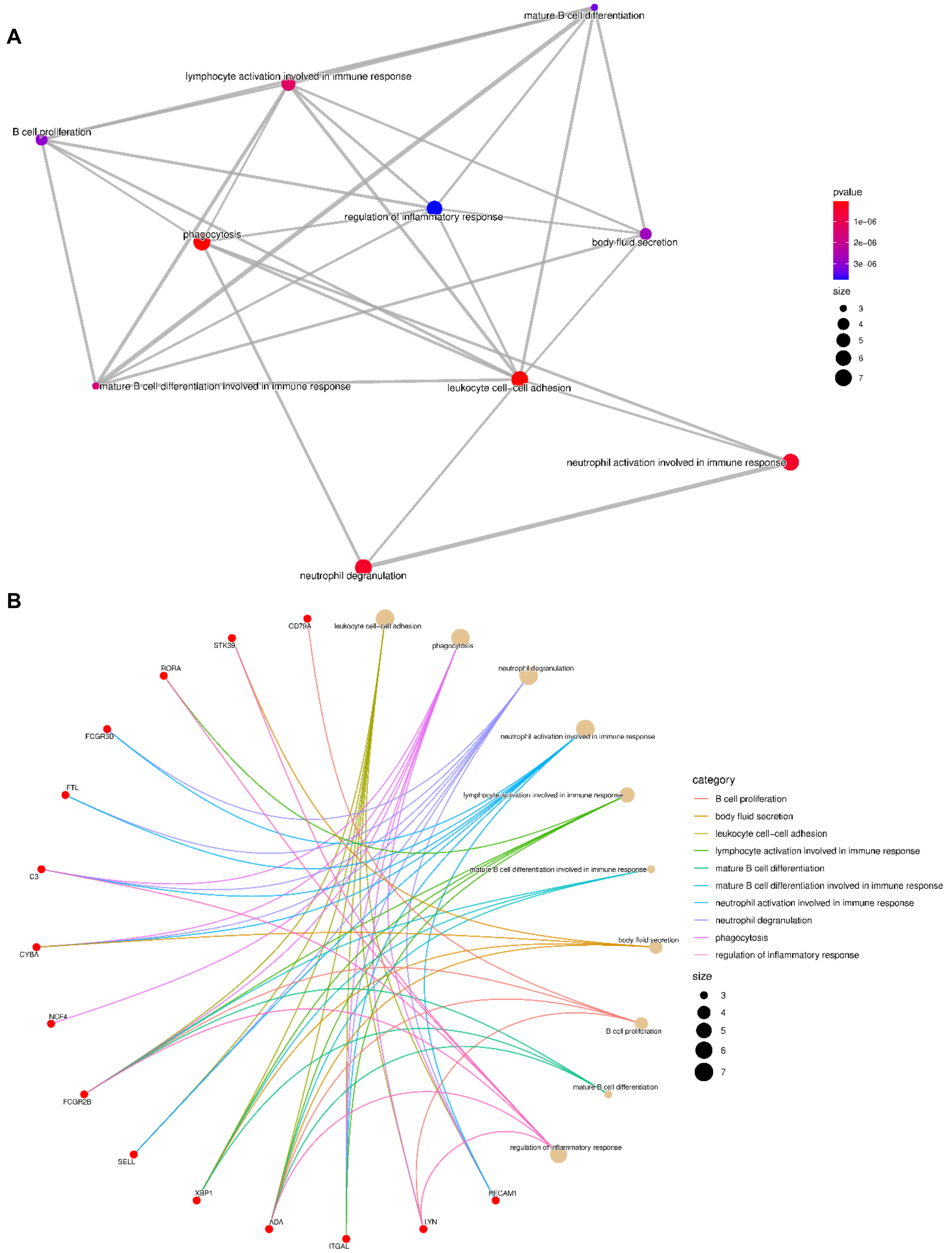

Supplementary Figure S6 (A) The relation of biological processes involved in top 20 paired genes; (B) The networks of top 20 paired genes with biological processes.

A

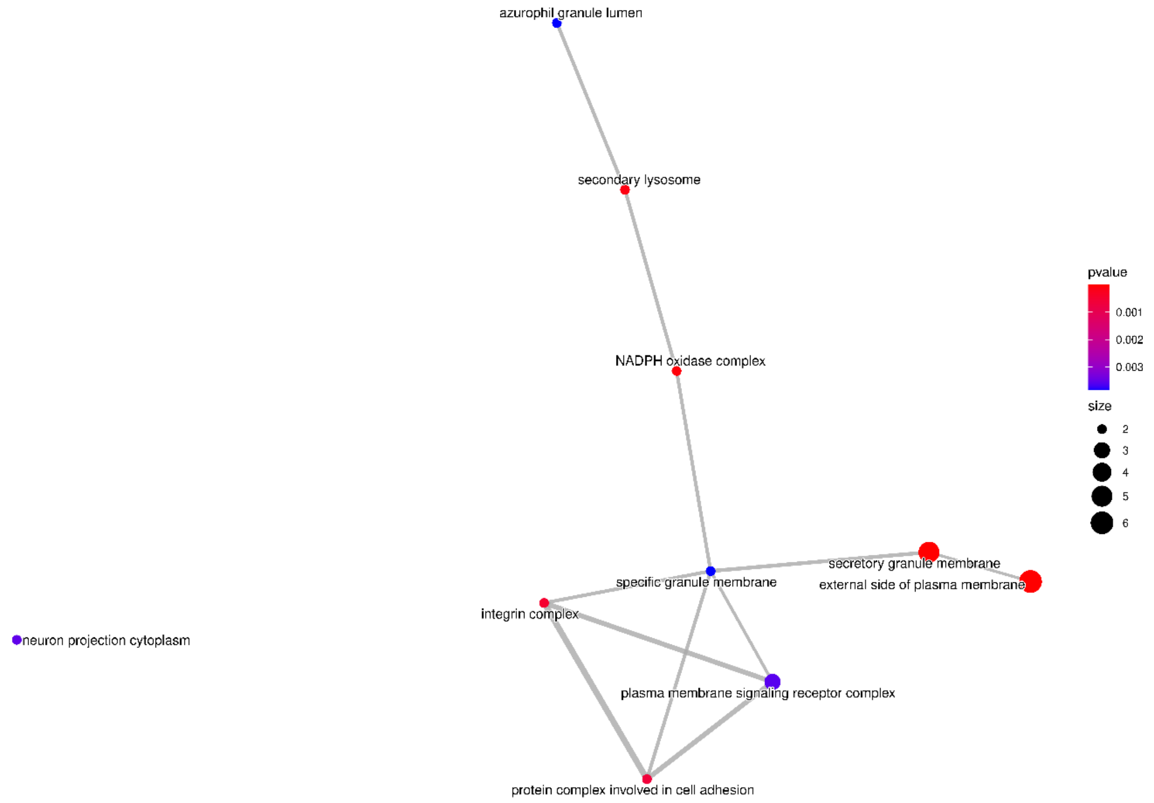

B

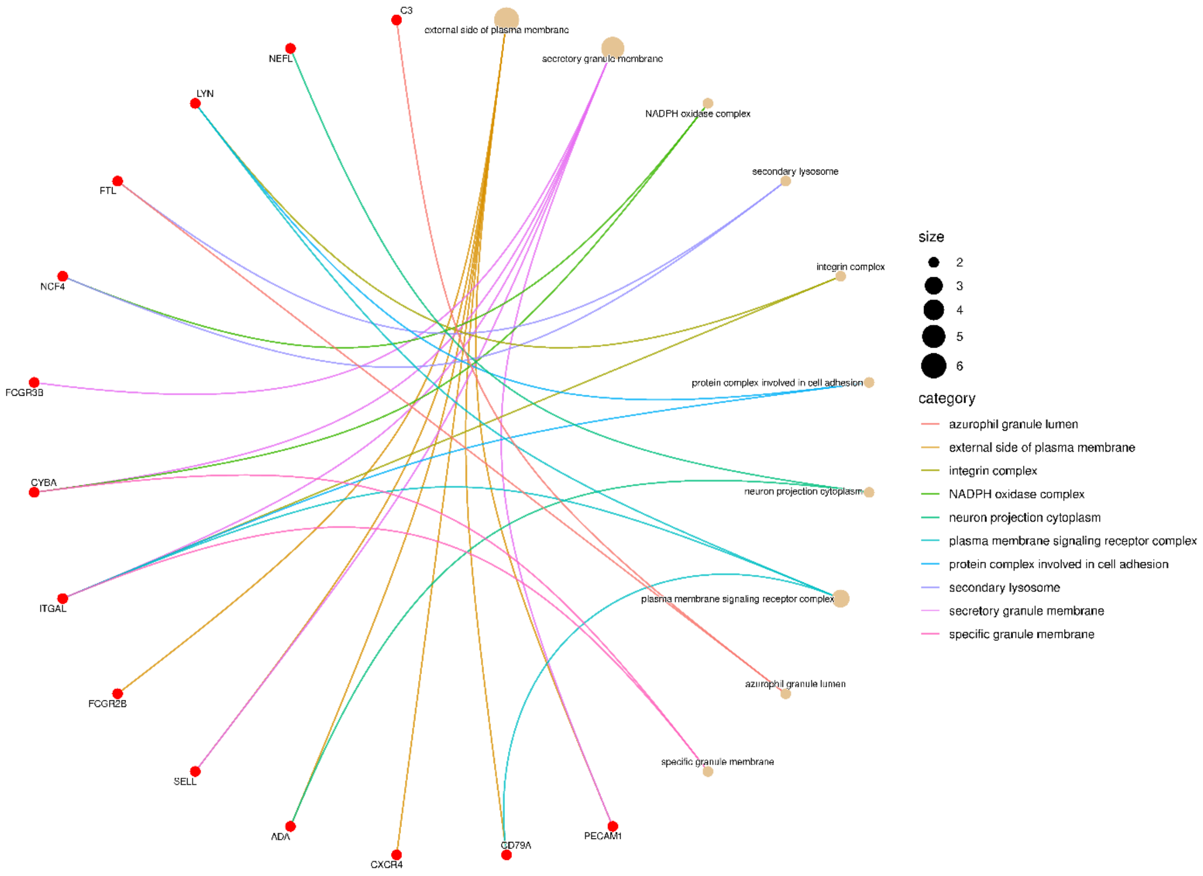

Supplementary Figure S7 (A) The relation of cellular components involved in top 20 paired genes; (B) The networks of top 20 paired genes with cellular components.

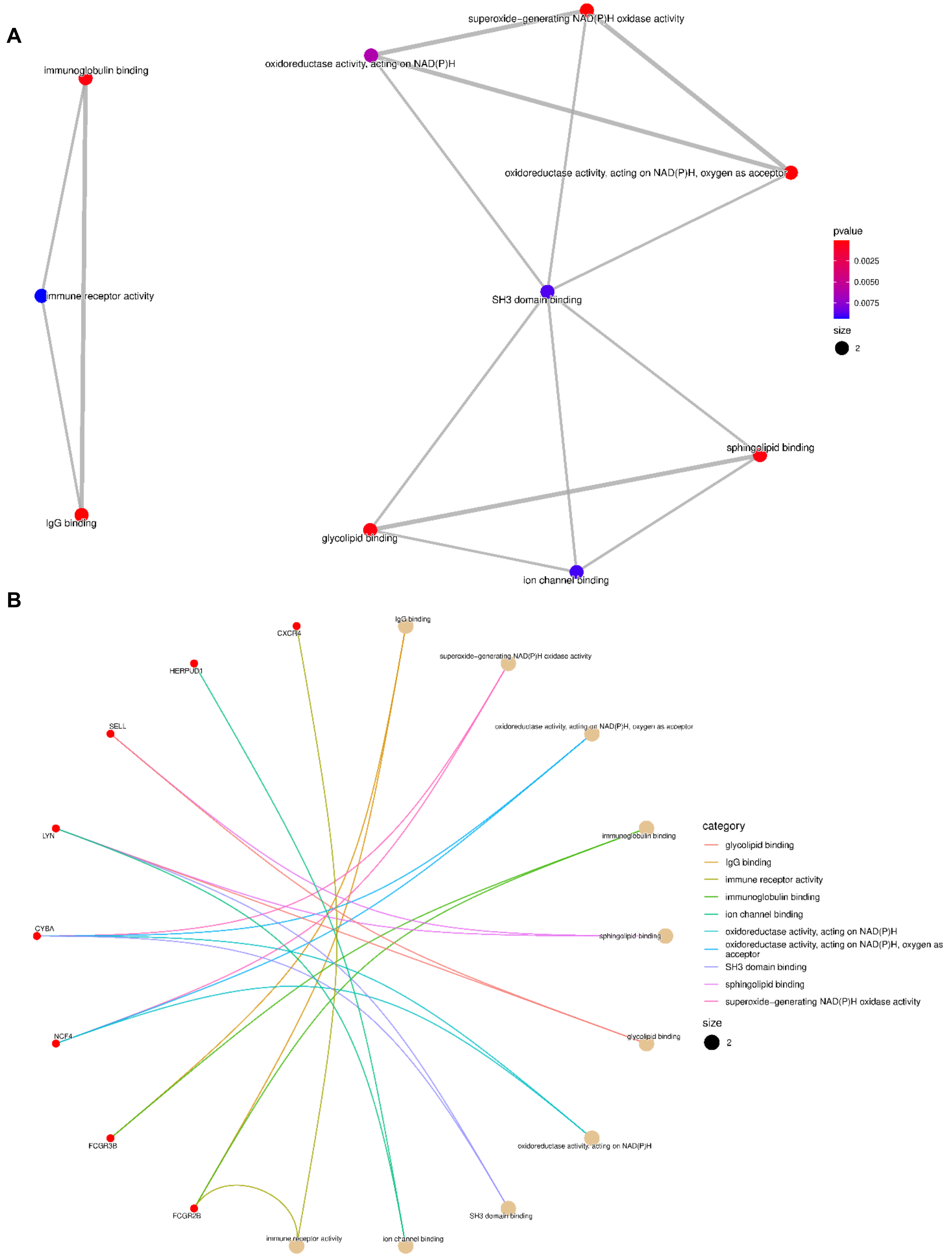

Supplementary Figure S8 (A) The relation of molecular functions involved in top 20 paired genes; (B) The networks of top 20 paired genes with molecular functions.

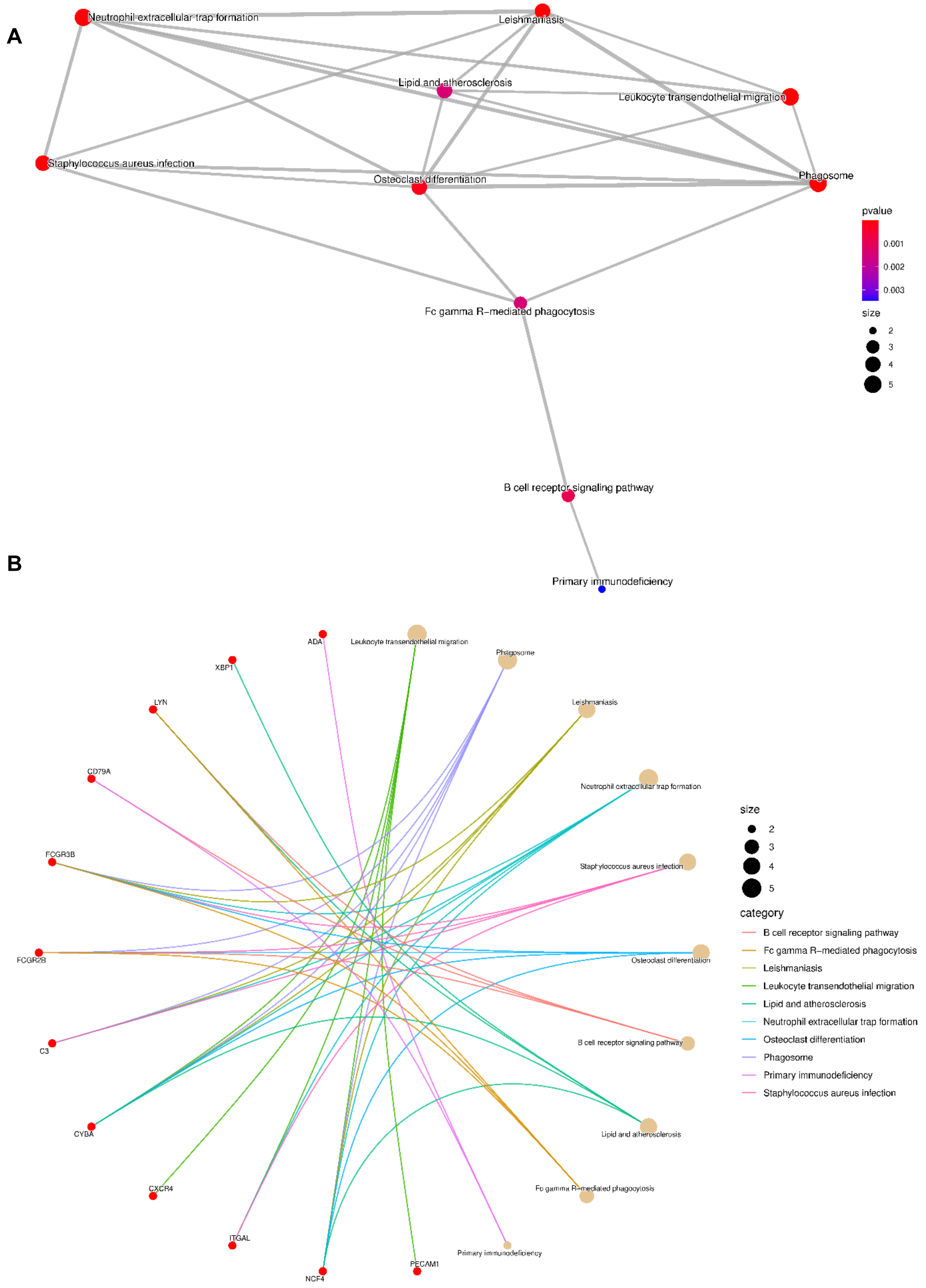

Supplementary Figure S9 (A) The relation of KEGG pathways involved in top 20 paired genes; (B) The networks of top 20 paired genes with KEGG pathways.

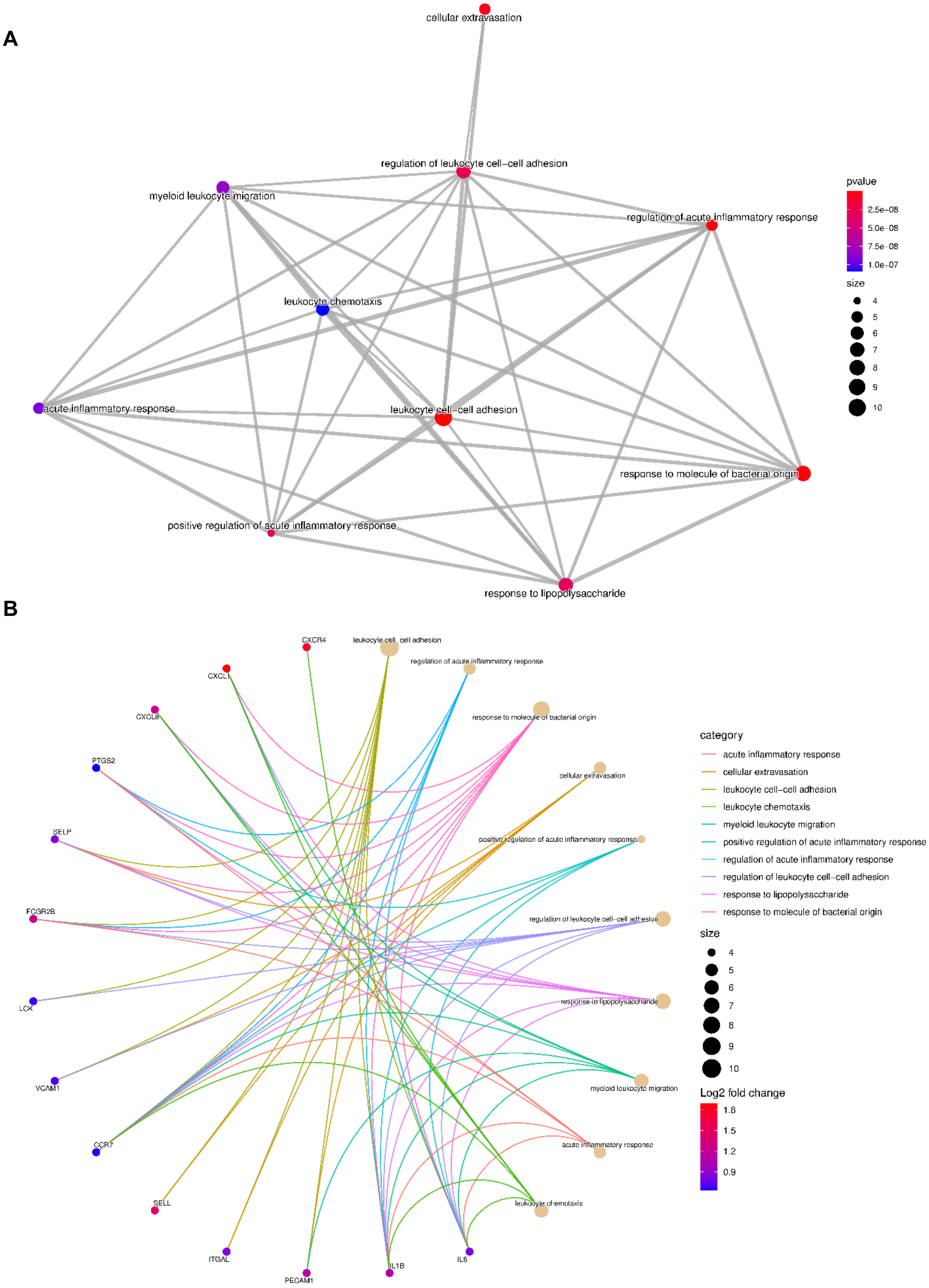

Supplementary Figure S10 (A) The relation of biological processes involved in hub OS-genes; (B) The networks of hub OS-genes with biological processes.

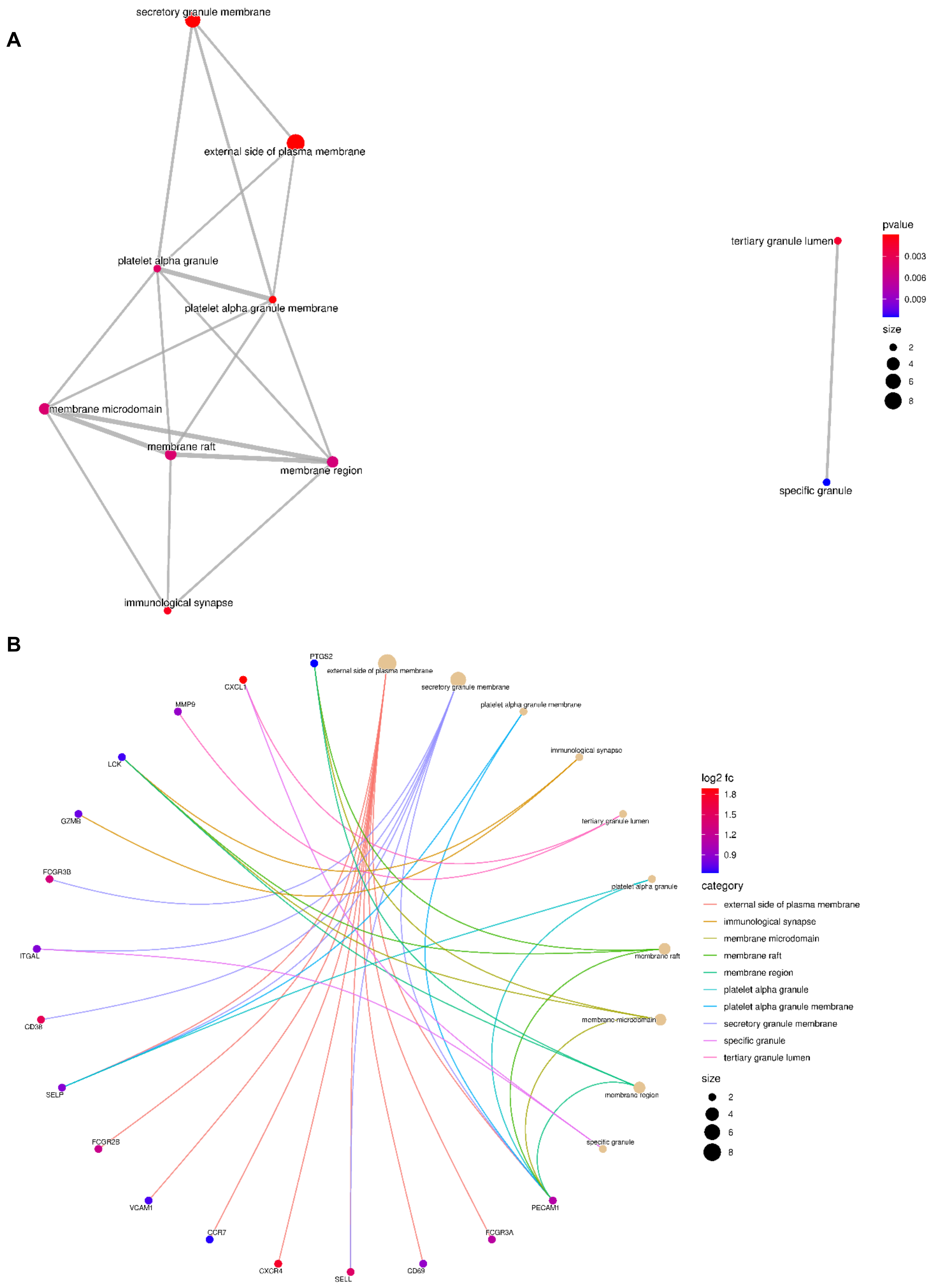

Supplementary Figure S11 (A) The relation of cellular components involved in hub OS-genes; (B) The networks of hub OS-genes with cellular components.

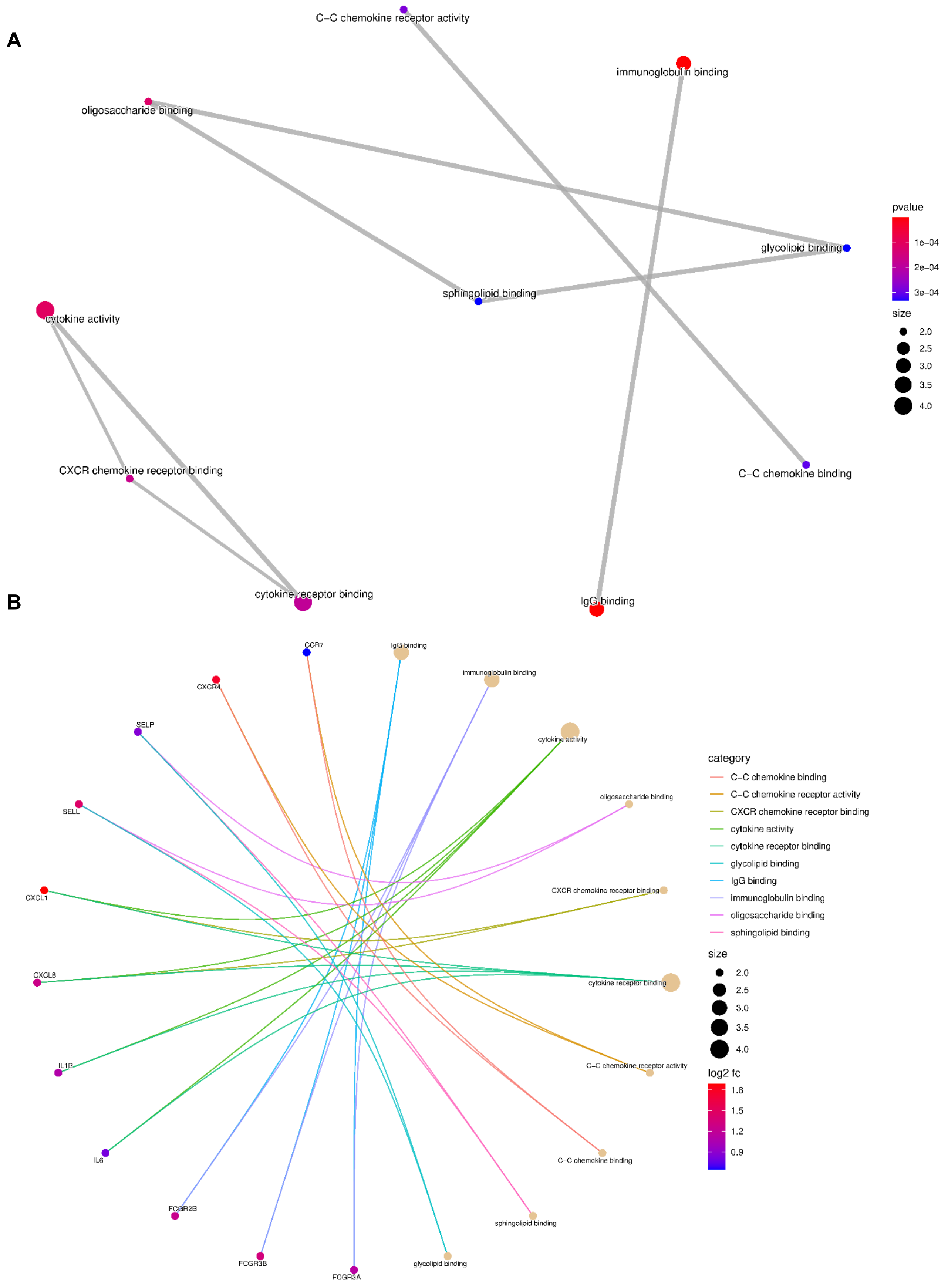

Supplementary Figure S12 (A) The relation of molecular functions involved in hub OS-genes; (B) The networks of hub OS-genes with molecular functions.

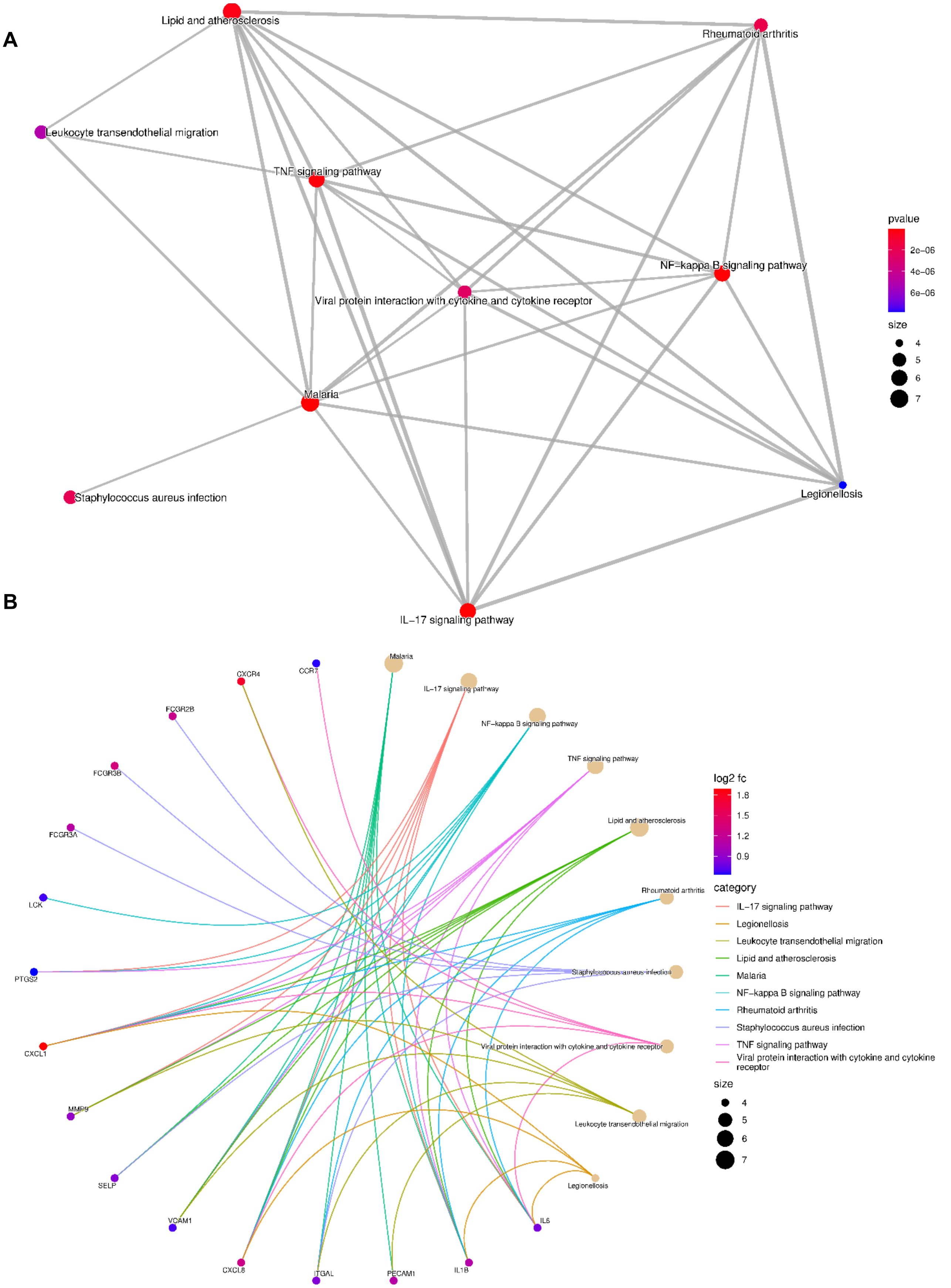

Supplementary Figure S13 (A) The relation of KEGG pathways involved in hub OS-genes; (B) The networks of hub OS-genes with KEGG pathways.
